# One-Year Clinical Success of Embrace Hydrophilic and Helioseal-F Hydrophobic Sealants in Permanent First Molars: A Clinical Trial

**Published:** 2017-03

**Authors:** Nahid Askarizadeh, Haleh Heshmat, Nazanin Zangeneh

**Affiliations:** 1 Associate Professor, Department of Pediatric, Dental Branch, Islamic Azad University, Tehran, Iran; 2 Assistant Professor, Department of Restorative Dentistry, Islamic Azad University, Tehran, Iran; 3 Pediatric Dental Resident, Department of Pediatric Dentistry, Dental Branch, Islamic Azad University, Tehran, Iran

**Keywords:** Pit and Fissure Sealants, Embrace Wetbond, Helioseal, Dentition, Permanent

## Abstract

**Objectives:**

This study sought to compare the one-year clinical success of a hydrophilic and a hydrophobic fissure sealant in permanent first molars.

**Materials and Methods:**

This split-mouth clinical trial was conducted on 23 six to nine year olds who had four fully erupted sound first molars. Helioseal-F and Embrace sealants were randomly applied on the first molars, and follow-ups were scheduled at three, six and 12 months to examine the teeth according to USPHS criteria (retention, marginal adaptation, color match, surface smoothness and caries recurrence). The Wilcoxon signed rank test, the Friedman test and the Mann Whitney test were applied for statistical analyses (P<0.05).

**Results:**

No significant differences were noted between Embrace and Helioseal-F in retention, smoothness of surface, marginal adaptation, color match or caries at three, six or 12 months (all P>0.05). In addition, the retention of sealants between the maxilla and mandible was not significantly different (P>0.05). Friedman test revealed no significant difference in any of the five parameters at different time points in any sealant group (P>0.05).

**Conclusions:**

Embrace hydrophilic and Helioseal-F hydrophobic sealants have the same one-year clinical success rate.

## INTRODUCTION

Fissure sealant treatment is an effective modality for prevention of occlusal caries [1,2]. However, some problems exist regarding the use of fissure sealants on erupting first molars considering their long eruption time, moisture-sensitivity of the conventional fissure sealants and deep pits and fissures of the newly erupted first molars [1–3]. Moreover, there are other challenges of using sealants such as their inability to penetrate well into the grooves, presence of gingival operculum distal to the teeth and insignificant effect of fluoride on occlusal pits and fissures [1–5]. Inadequate isolation and saliva contamination during the procedure are the main reasons for failure of fissure sealants in their first year of application [6,7]. The conventional resin sealants require an isolated, completely dry work field for their clinical success; this decreases their success rate in semi-erupted permanent first molars since complete isolation of such teeth is difficult [7,8]. Efficient cleaning of occlusal grooves, application of a low viscosity hydrophilic bonding agent beneath the sealant and complete isolation of tooth improve the efficacy of sealants [7,9].

Recently, some modifications were made in chemical formulation of sealants [6,7,10]. Resin sealants are bonded to the underlying enamel using the acid-etching technique [11]. By creating a physical barrier as such, metabolic communication between the microorganisms present in the occlusal grooves and oral environment is prevented [1,2]. Although glass ionomers and composite resins are extensively used in dental procedures, their main shortcoming is their moisture susceptibility. Even in case of complete drying of tooth, some moisture still remains in the tooth structure [3,6, 7,10,12]. Embrace is a moisture-resistant resin sealant, which does not contain bisphenol A or bis-GMA [5,6]. It has a hydrophilic-base and contains di-tri-acrylate and multi-functional monomers. It also contains 36.6% filler particles, which are activated by moisture [5–7]. It has the advantages of providing an optimal bond in wet environment, optimal retention, high marginal adaptation, smooth margins, low technical sensitivity and fluoride release potential [2,5–7, 10,12]. After curing, its acidic pH becomes neutral, making it almost insoluble in water [1, 5, 7]. Due to optimal properties, this sealant has been suggested for use in uncooperative children and cases of difficult isolation [6,7]. Several previous studies have compared Embrace hydrophilic sealant with the conventional hydrophobic sealants, yielding controversial results [1,2,5–7,10]. Some of them have reported higher success rate for hydrophilic sealant [5,6] while some others did not find any significant difference between the two types [1,2,7]. Another study showed inadequate retention of Embrace [10].

Considering the existing controversy in this respect, this study sought to compare the one-year clinical success of Embrace hydrophilic and Helioseal-F hydrophobic sealants in permanent first molars of children aged six to nine years.

## MATERIALS AND METHODS

This split-mouth randomized single blind clinical trial was conducted on 23 cooperative children between six to nine years [2,9,13] who were rated 3 or 4 using the Frankel’s behavior rating scale. They were randomly selected from those presenting to a private dental clinic requiring fissure sealant treatment of four permanent first molars. The study protocol was approved in the ethics committee of Islamic Azad University, School of Dentistry (code:104) and registered in www.irct.ir (code: IRCT2014072618601N1). Written informed consent was obtained from the parents or legal guardians of children.

Sample size was calculated to be 30 permanent first molars in each group (a total of 60) based on a previous study [10]. Considering the loss to follow-up, 46 teeth in each group (total of 92) were required and since four permanent molars of each patient were included, 23 children who met the inclusion criteria were recruited.

The inclusion criteria were: Presence of four fully-erupted permanent first molars with clearly visible occlusal surface [1,4,12] and deep pits and fissures [1,4,6,10] without occlusal or proximal caries [4,10], absence of hypocalcification or hypoplasia in the respective teeth [1,5–7], no history of previous treatment on the respective teeth [5,10] (these criteria were evaluated by clinical examination of the teeth using a mirror and a dental explorer as well as on bitewing radiographs, which were fully inspected by two pediatric dentists), possibility of acceptable isolation by use of cotton rolls [5,11], no remarkable medical history [5,6,10], not taking drugs affecting the salivary flow such as tricyclic anti-depressants, atropine, anti-histamines, diuretics or bronchodilators [10,14], no allergy to any restorative material [13,15] (determined by asking the parents) and low risk of caries (determined by caries risk assessment) [2,13,15].

The study had a split-mouth design. In the first appointment, all children received oral hygiene instructions including proper use of dental floss, tooth brush and toothpaste, and the need for fissure sealant treatment of all four permanent first molars was confirmed by clinical examination using a dental mirror and a dental explorer (Atlasdent, Tehran, Iran). In the next appointment, the children were requested to brush their teeth to ensure the accuracy of tooth brushing technique. Dental prophylaxis and cleaning were done for all teeth using a low-speed hand piece and a prophy brush (Atlasdent, Tehran, Iran) to remove debris from the grooves. The occlusal surfaces of the teeth were rinsed with water and checked by an explorer. Permanent first molars were isolated using cotton rolls and saliva ejector. Allocation of type of sealant to the right/left quadrant of the maxilla/mandible was done randomly by flipping a coin. Sealant was applied by a second-year post-graduate student of pediatric dentistry under the supervision of a pediatric dentist.

In group one, the occlusal surface of the teeth was dried and etched with 37% phosphoric acid (Ivoclar Vivadent AG, Schaan, Liechtenstein) for 15 seconds according to the manufacturer’s instructions, rinsed for 30 seconds and air-dried with oil- and water-free air spray. Under cotton roll isolation and upon observing the chalky white appearance, Helioseal-F sealant (Ivoclar Vivadent AG, Schaan, Liechtenstein) was applied and directed into the pits and fissures by a sterile explorer and light cured for 40 seconds by a light-curing unit (Monitex, Tokyo, Japan) with a light intensity of 400mW/cm^2^.

In group two, the occlusal surface of the teeth was dried and etched with 37% phosphoric acid (Ivoclar Vivadent AG, Schaan, Liechtenstein) for 20 seconds according to the manufacturer’s instructions, rinsed for 30 seconds and gently dried with cotton rolls in such a way that the occlusal surface had a shiny appearance. Embrace WetBond (Pulpdent Co., Watertown, USA) was applied and directed into the pits and fissures by a sterile explorer and light cured as described for group one. Next, the occlusion was checked in both groups (both sides) using an articulating paper, and occlusal interferences were eliminated. All phases were carried out according to the manufacturers’ instructions. Follow-up sessions were scheduled at three, six and 12 months for assessment of sealants [1,2, 13,15]. In each follow-up session, the fissure sealants were clinically examined by a pediatric dentist and a second-year post-graduate student of pediatric dentistry using a dental mirror and an explorer [6,7,10]. One week after the first clinical examination, 10 patients were randomly selected and examined again. The inter- and intra-observer agreements (kappa values) were found to be 86% and 89%, respectively. The quality of fissure sealants was assessed using the modified Ryge (USPHS) criteria [16], which assess retention, marginal adaptation, color match, smoothness of surface and caries recurrence [14,16]. Table 1 shows the method of classification of each of these parameters.

**Table 1.** Assessment of clinical success of fissure sealants using modified Ryge criteria (USPHS)

**Criterion**	**Grade**	**Definition**
Retention	A	Sealant was intact
	B	Sealant was partially lost
	C	Sealant was totally lost

Caries	A	Absence of caries
	B	Presence of caries

Marginal adaptation	A	Sealant had full adaptation to the adjacent tooth structure
	B	Evidence of gap detected by the tip of an explorer

Color match	A	Acceptable color match
	B	Slight discoloration

Smoothness of surface	A	As smooth as the adjacent tooth structure
	B	Not as smooth as the adjacent tooth structure but not porous
	C	Not as smooth as the adjacent tooth structure and porous

The data were analyzed using SPSS version 22 (SPSS Inc., IL, USA). The Wilcoxon signed rank test was applied to compare the clinical success criteria at each time point between the two materials. Friedman test was used for intragroup comparisons between different time points. The Mann Whitney test was used to compare retention between the maxilla and mandible. P<0.05 was considered statistically significant.

## RESULTS

This study was performed on 23 children between six to nine years with a mean age of 7.61±0.881 years; out of which, 13 were girls and 10 were boys. A total of 92 permanent first molars (46 maxillary and 36 mandibular) were evaluated in a split-mouth design.

Two patients were excluded at the six-month and one at the 12-month follow-up. Tables 2, 3 and 4 present the results of assessment of the success criteria at three, six and 12 months, respectively in the two groups.

**Table 2:** Assessment of success criteria in the two groups at three months

**Groups/criteria**	**Retention**			**Color match**		**Marginal adaptation**		**Surface smoothness**			**Caries**	

	**Complete N(%)**	**Partial N(%)**	**Absent N(%)**	**Yes N(%)**	**No N(%)**	**Yes N(%)**	**No N(%)**	**Normal N(%)**	**Abnormal N(%)**	**Cavitated N(%)**	**Yes N(%)**	**No N(%)**
**Helioseal-F**	43(93.5)	3(6.5)	0(0)	46(100)	0(0)	43(93.5)	3(6.5)	44(95.7)	2(4.3)	0(0)	0(0)	46(100)
**Embrace**	44(95.7)	2(4.3)	0(0)	46(100)	0(0)	44(95.7)	2(4.3)	43(93.5)	3(6.5)	0(0)	0(0)	46(100)

**P value**	P=0.655			P=1.000		P=0.655		P=0.655			P=1.000	

**Table 3:** Assessment of success criteria in the two groups at six months

**Groups/criteria**	**Retention**			**Color match**		**Marginal adaptation**		**Surface smoothness**			**Caries**	

	**Complete N(%)**	**Partial N(%)**	**Absent N(%)**	**Yes N(%)**	**No N(%)**	**Yes N(%)**	**No N(%)**	**Normal N(%)**	**Abnormal N(%)**	**Cavitated N(%)**	**Yes N(%)**	**No N(%)**
**Helioseal-F**	34(81)	8(19)	0(0)	40(95.2)	2(4.8)	34(81)	8(19)	36(85.7)	6(14.3)	0(0)	0(0)	42(100)
**Embrace**	35(83.3)	7(16.7)	0(0)	40(95.2)	2(4.8)	35(83.3)	7(16.7)	34(81)	8(19)	0(0)	0(0)	42(100)

**P value**	P=0.763			P=1.000		P=0.763		P=0.527			P=1.000	

**Table 4:** Assessment of success criteria in the two groups at 12 months

**Groups/criteria**	**Retention**			**Color match**		**Marginal adaptation**		**Surface smoothness**			**Caries**	

	**Complete N(%)**	**Partial N(%)**	**Absent N(%)**	**Yes N(%)**	**No N(%)**	**Yes N(%)**	**No N(%)**	**Normal N(%)**	**Abnormal N(%)**	**Cavitated N(%)**	**Yes N(%)**	**No N(%)**
**Helioseal-F**	25(62.5)	12(30)	3(7.5)	33(82.5)	7(17.5)	25(62.5)	15(37.5)	29(72.5)	8(20)	3(7.5)	1(2.5)	39(97.5)
**Embrace**	24(60)	14(35)	2(5)	31(77.5)	9(22.5)	24(60)	16(40)	27(67.5)	11(27.5)	2(5)	3(7.5)	37(92.5)

**P value**	P=1.000			P=0.527		P=0.841		P=0.846			P=0.317	

In terms of retention, no significant difference was noted at the three, six and 12-month follow-ups between the two sealants. At 12 months, 62.5% of Embrace and 60% of Helioseal-F cases had complete retention (P=1.000).

No significant difference was noted in terms of smoothness of surface between the two groups. At 12 months, 32.5% of Embrace and 27.5% of Helioseal-F sealants lost their surface smoothness (P=0.846). With regard to color match, 82.5% of teeth in Embrace and 77.5% of those in Helioseal-F group still had optimal color match at one year (P=0.527). Marginal adaptation was not significantly different either between the two groups at any time point. At 12 months, 60% of teeth in Embrace and 62.5% in Helioseal-F group had acceptable marginal adaptation (P=0.841). At the 12-month follow-up, three teeth in Embrace and one tooth in Helioseal-F group were carious; this difference between the two groups was not significant (P=0.317). Friedman test showed that the two groups were not significantly different in terms of the five success criteria (altogether; P>0.05). No significant differences were noted in loss of retention of sealants between the maxilla and mandible, between the two types of sealants at each time point or among different time points (all P>0.05).

The difference in retention of sealants in the occlusal, palatal or buccal fissures was not significant either at any time point (P>0.05).

## DISCUSSION

This study assessed and compared the clinical success of Embrace hydrophilic and Helioseal-F hydrophobic sealants in 23 permanent fist molars of children between six to nine years in a split mouth design using modified Ryge (USPHS) criteria at three, six and 12 months. The results showed no significant difference between the two sealants in any of the five assessed success criteria. Fissure sealants prevent the penetration of microorganisms, foods and saliva into the pits and fissures and inhibit subsequent development of caries [6]. Occlusal fissures are eight times more susceptible to caries than smooth surfaces [6,17]. Thus, sealant retention is especially important. The conventional resin sealants have high technical sensitivity and their clinical success is affected by patient cooperation, contamination of the area and expertise and skills of the operator [6,7,10]. However, moisture susceptibility complicates sealant therapy in semi-erupted teeth and uncooperative children [3,6,12]. Risk of occlusal caries is the highest in the first years following eruption of teeth because the enamel is slightly porous and the grooves are full of cellular and organic debris.

Risk of occlusal caries is the highest in the first years following eruption of teeth because the enamel is slightly porous and the grooves are full of cellular and organic debris. Thus, it is important to find a sealant with high clinical success rate for use in semi-erupted molars, which are difficult to isolate [6,18].

The manufacturer claims that Embrace sealant is self-priming and self-adhesive and has low technical sensitivity. It is activated with moisture and releases fluoride. It also chemically bonds to tooth structure [4,5]. It is devoid of bis-GMA and bisphenol A. It is important in that bis-GMA tends to bond to estrogen receptors [6,18]. Most composite resins and sealants have bis-GMA and bisphenol A diglycidyl ether methacrylate in their composition. Evidence shows that complete polymerization does not occur in these compounds, and free monomers are detectable in the saliva. Bisphenol A and aromatic compounds react with biological molecules and bond to estrogen receptors [19].

Our results regarding lack of a significant difference in the clinical success of the two sealants was in line with those of Bhatia et al, [1] Bhat et al, [2] and Subramaniam et al [7]. In the study by Bhatia et al, [1] retention of Embrace was slightly superior to that of Delton conventional sealant. They explained that the efficacy of sealants mainly depends on the clinical procedural steps and quality of the material.

They used Simonsen’s criteria for assessment of retention; but retention alone cannot be the only indicator of clinical success of a sealant. Material properties such as solubility are very important in clinical success of restorations and fissure sealants. High solubility of a dental material results in its degradation and decreases the longevity of restoration [20]. Filler content, filler size, filler surface area, type of filler particles and degree of polymerization all affect the solubility of materials. Voids created during application or mixing prevent polymerization and increase solubility of materials [21]. Thus, the procedural steps and technical sensitivity of sealants have a direct effect on their stability. Moreover, some researchers believe that compounds containing bis-GMA have lower solubility than compounds devoid of it or those containing UDMA [22].

Water sorption also affects the stability and retention of fissure sealants. Presence of hydrophilic compounds such as HEMA results in greater water sorption [23]. Thus, Embrace is expected to have higher water sorption due to its hydrophilicity. However, clinical success of a restoration in the oral environment is the result of interaction of several factors including physicochemical properties, method of application and technical sensitivity. For fissure sealants, isolation is very important, and materials providing an acceptable wet bond are preferred. In vitro studies have shown that Embrace has a higher tensile strength than resin cements [10]. Also, it has lower viscosity, forms longer resin tags, provides high marginal adaptation and well penetrates into deep grooves compared to bis-GMA sealants [24,25]. Furthermore, due to its hydrophilicity, Embrace has lower technical sensitivity than Helioseal-F [7,8,10].

Embrace is acidic before curing. After light curing, it has a neutral pH with physicochemical properties similar to those of conventional sealants. In presence of moisture, it flows on the surface and bonds to tooth structure [7]. Thus, it does not require a bonding agent; whereas, hydrophobic (conventional) sealants do not bond to wet surfaces [7]. Despite lower technical sensitivity of Embrace than Helioseal F, they both have similar wear, water sorption, solubility and retention [5]. Therefore, in case of difficult isolation (uncooperative patients, those with physical or mental disabilities, semi-erupted molars, etc.), Embrace is recommended for fissure sealant treatment [6]. In the study by Bhat et al, [2] Embrace was not significantly different from two conventional sealants in terms of retention but it showed significantly higher retention than glass ionomer, which is probably due to the weaker bond of glass ionomer to the enamel.

Subramaniam et al. [7] found results similar to ours using modified CCC criteria, which is a visual-tactile index that assesses several clinical criteria including discoloration, caries and surface texture of sealant determined by use of a periodontal probe. In a study by Ratnaditya et al, [6] retention of Embrace was significantly higher than that of Delton after two years. They used Simonsen’s criteria for clinical assessment of retention of fissure sealants. Moreover, retention of Embrace in mandibular teeth was greater than that in maxillary teeth. They explained the reason to be better visibility of mandibular teeth and the gravity. They mentioned that occlusal stress affects sealants in maxillary molars earlier after eruption compared to mandibular molars [4,6]. However, in our study, no significant difference was noted in retention of sealants between the maxilla and mandible. We believe that sealant retention is not influenced by the maxillary or mandibular arch if a good-quality sealant is properly applied.

In contrast, Schlueter et al. [10] showed significantly lower retention of Embrace than Helioseal-F at one year. They used the clinical index of retention and sealant quality, which included air inclusion, marginal adaptation, marginal discoloration and pit and fissure caries. They found no significant difference in retention of sealants between the maxilla and mandible. Use of different etching times for the two sealants may be responsible for their different retention. They attributed the lower success rate of embrace to difficult moisture control in grooves since the grooves should not be too dry (to see the chalky white appearance) or too wet (to see water droplets) and this is hard to achieve in the clinical setting. The other reason was assumed to be water sorption by Embrace and subsequently increased solubility and higher risk of disintegration [10].

Smoothness of the surface was a success criterion evaluated in the current study, which indicates wear resistance of the material. The two sealants showed similar smoothness, which indicates similar wear resistance [13]. Another criterion was marginal adaptation. Poor marginal adaptation may be due to incomplete polymerization of sealant, inadequate light intensity of the light-curing unit or water sorption. In the current study, change in marginal adaptation was minimal in both groups.

Erroneous technique (i.e. working in wet environment) and wear due to occlusal loads are the two main reasons for failure and loss of fissure sealants [7]. The two sealants were not significantly different in terms of caries in our study, which may be due to fluoride uptake by the adjacent enamel. Even in case of loss of sealant, the rest of the sealant often remains in the grooves and serves its protective role [4,13,15].

Occlusal surfaces of the first and second molars have the highest risk of occlusal caries [5]. In the current study, both sealants were equal in terms of caries prevention. The allocation of sealants to jaws and quadrants was random to prevent bias [7]. The follow-ups were scheduled at three, six, nine and 12 months. Some cases of failure occurred as early as three months, which indicates that an appointment must be scheduled for patients as early as three months. Also, since retention showed a descending trend over time, a 12-month follow-up is also required.

The new generation of fissure sealants containing hydrophilic monomers appears to have lower technical sensitivity since it is moisture-tolerant. Also, it does not require a bonding agent for wet bonding. It decreases the treatment time and enhances patient cooperation [8]. Future studies with longer follow-ups are required to assess the performance of these sealants over longer periods of time. Moreover, other properties of Embrace such as its fluoride release potential, water sorption, microleakage and compressive strength must be evaluated in future in vitro studies.

## CONCLUSION

Within the limitations of this study, the results showed that the one-year clinical success of Embrace hydrophilic sealant was similar to that of Helioseal-F hydrophobic sealant.
